# Intracellular Competitions Reveal Determinants of Plasmid Evolutionary Success

**DOI:** 10.3389/fmicb.2020.02062

**Published:** 2020-09-04

**Authors:** Nils F. Hülter, Tanita Wein, Johannes Effe, Ana Garoña, Tal Dagan

**Affiliations:** Institute of General Microbiology, Kiel University, Kiel, Germany

**Keywords:** genome evolution, lateral gene transfer, antibiotics resistance, extrachromosomal DNA, homologous recombination

## Abstract

Plasmids are autonomously replicating genetic elements that are ubiquitous in all taxa and habitats where they constitute an integral part of microbial genomes. The stable inheritance of plasmids depends on their segregation during cell division and their long-term persistence in a host population is thought to largely depend on their impact on the host fitness. Nonetheless, many plasmids found in nature are lacking a clear trait that is advantageous to their host; the determinants of plasmid evolutionary success in the absence of plasmid benefit to the host remain understudied. Here we show that stable plasmid inheritance is an important determinant of plasmid evolutionary success. Borrowing terminology from evolutionary biology of cellular living forms, we hypothesize that Darwinian fitness is key for the plasmid evolutionary success. Performing intracellular plasmid competitions between non-mobile plasmids enables us to compare the evolutionary success of plasmid genotypes within the host, i.e., the plasmid fitness. Intracellular head-to-head competitions between stable and unstable variants of the same model plasmid revealed that the stable plasmid variant has a higher fitness in comparison to the unstable plasmid. Preemptive plasmid competitions reveal that plasmid fitness may depend on the order of plasmid arrival in the host. Competitions between plasmids characterized by similar stability of inheritance reveal plasmid fitness differences depending on the plasmid-encoded trait. Our results further reveal that competing plasmids can be maintained in coexistence following plasmid fusions that maintain unstable plasmid variants over time. Plasmids are not only useful accessory genetic elements to their host but they are also evolving and replicating entities, similarly to cellular living forms. There is a clear link between plasmid genetics and plasmid evolutionary success – hence plasmids are evolving entities whose fitness is quantifiable.

## Introduction

Extrachromosomal genetic elements are ubiquitous in prokaryotes and eukaryotes. Viruses, mitochondria, bacteriophages and plasmids are examples for extrachromosomal genetic elements that depend on a hosting cell for reproduction where they replicate independently of the chromosome. Some elements are beneficial for their host (e.g., eukaryotic organelles), others are characterized as parasitic (e.g., viruses) or selfish (e.g., transposons). It is commonly agreed that extrachromosomal elements evolve by ‘descent with modification’ where genetic modifications are heritable over time and generations. Nonetheless, studying the effects of natural selection on the evolution of extrachromosomal elements is a challenging concept; how to disentangle the evolutionary success of extrachromosomal elements from that of their host remains an open question in evolutionary biology.

Here we focus on prokaryotic plasmids, which have been extensively studied due to their contribution to gene transfer during microbial evolution. Plasmids encoding for resistance mechanisms to antibiotics confer an immediate advantage to their host fitness under selection for antibiotics resistance. Such plasmids are often found in ecosystems exposed to fluctuating levels of antibiotics (e.g., Pham et al., 2018; González-Plaza et al., 2019). Nonetheless, many plasmids found in nature are lacking a clear trait that is advantageous to the host (i.e., ‘cryptic’ plasmids). For example, a recent study of the rat cecum meta-mobilome revealed hundreds of small novel cryptic plasmids (Jørgensen et al., 2014). Such small cryptic plasmids may carry very few genes next to their replication machinery (e.g., Carneiro et al., 2016; Zhou et al., 2018). Plasmid persistence (i.e., the plasmid presence within a microbial population over time) in the absence of positive selection is mainly dependent on stable plasmid replication and segregation, that is, reproduction and vertical inheritance (Bergstrom et al., 2000; Paulsson, 2002). An important property of plasmids is the number of plasmid copies in the cell (plasmid copy number; PCN) that is tightly regulated by copy number control mechanisms encoded by the plasmid (e.g., Pinkney et al., 1988; Kittell and Helinski, 1991; Wagner et al., 1992). Plasmid replicons are thus typically polyploid hence the plasmid population within a host cell may comprise multiple plasmid variants (i.e., alleles) due to independent emergence of mutations in the plasmid copies (Bedhomme et al., 2017; Rodriguez-Beltran et al., 2018). The PCN can be variable among cells in both space and time and it has important implications for the segregation of plasmid variants (Ilhan et al., 2019). Plasmid segregation into daughter cells during cell division may be facilitated by active partition mechanisms (Baxter and Funnell, 2014) or plasmid diffusion in the cell (Wang, 2017).

Plasmids whose inheritance is unstable may persist in the population, e.g., via infective transmission to plasmid-free cells (Heuer et al., 2007; Hall et al., 2016). Additionally, plasmids may carry functions that confer a competitive advantage to their host, and thus ensure that plasmid-carrying cells will out-compete plasmid-free cells in the population. This includes functions that are beneficial for the host depending on the environmental conditions [e.g., *Lactococcus lactis* (McKay et al., 1976)], functions that are indispensible for the host regardless of the environmental conditions [e.g., symbiotic lifestyle maintenance (Grote et al., 2016)], and mechanisms for post-segregational killing of plasmid-free cells (Gerdes et al., 1986a, b), which may also lead to the exclusion of competing plasmids (Cooper and Heinemann, 2000). Other factors that may influence plasmid persistence in the population range from positive or negative interactions with the host cell, e.g., with the host replisome (Sota et al., 2010) or defense mechanisms (e.g., Mahendra et al., 2020), to the transcriptional load of plasmid genes (e.g., Buckner et al., 2018).

Previous studies on the evolution of plasmid persistence in naïve hosts focused mainly on plasmids with a large genome size (e.g., >50 Kb) that encode multiple functions that support the stable plasmid inheritance or the plasmid persistence in the population (e.g., Yano et al., 2016; Bottery et al., 2017; Loftie-Eaton et al., 2017). In contrast, small plasmids that do not confer a clear function to their host remain largely understudied. Plasmids that have a negligible effect on their host fitness may evolve a stable inheritance and thus become permanent in the population and an integral component of the lineage genome (Wein et al., 2019). However, quantifying plasmid evolutionary success requires us to disentangle the plasmid reproductive and segregation success from that of the host.

Borrowing terminology from evolutionary research of cellular living forms, we hypothesize that *Darwinian fitness* is key for the plasmid evolutionary success. Here we define Darwinian fitness of plasmids as the average contribution of a specific plasmid genotype to the plasmid allele pool in the next generation. The fitness of plasmids thus relies on their reproduction, i.e., replication in the cell, and in addition, on their segregation – i.e., inheritance during cell division. We further propose that the concept of plasmid fitness is key for understanding the existence and evolution of plasmids and other extra-chromosomal genetic elements. Notably, natural selection operates on plasmids in two hierarchical levels; one component of plasmid fitness is the host fitness within the population and the second component is the plasmid fitness within the cell. While the first component has been extensively studied, the second component has been so far largely neglected. Here we present a novel approach to compare the fitness of naturally occurring plasmid backbones within the host cell. Our approach includes pairwise *in cellulo* competitions between selected variants of the same plasmid backbone. We hypothesize that stable plasmid inheritance is an important determinant of plasmid fitness hence a plasmid variant characterized by stable inheritance is expected to outcompete a plasmid variant whose inheritance is unstable.

## Results

### Comparison of Plasmid Fitness Independent of the Bacterial Host Cell

**FIGURE 1:**
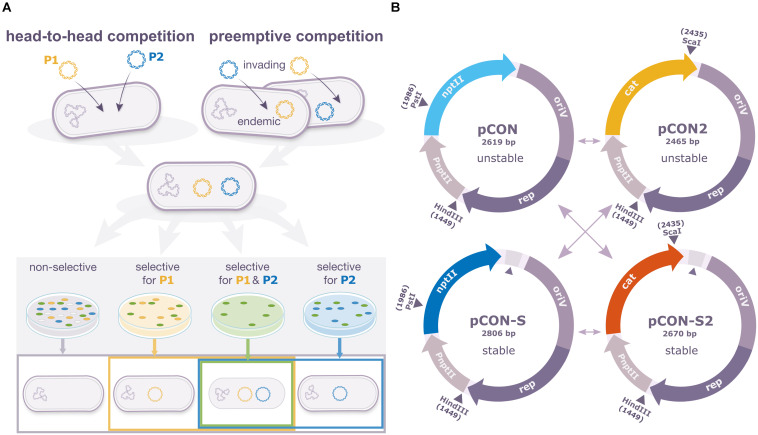
*In cellulo* comparison of plasmid fitness using pairwise competition experiments. **(A)** Pairwise competition assay designs. In the head-to-head competition mode, the competing plasmids are introduced into the host simultaneously. In the preemptive competition mode, the competing plasmids are introduced into the host consecutively. Hosts of both plasmids constitute the initial population for the competition. The result of the plasmid competition is evaluated according to the frequency of host types at the end of the experiments. These include plasmid-specific hosts, hosts of both plasmids in coexistence (as well as plasmid-free segregants). **(B)** Our set of competitor model plasmids comprised two unstable (pCON and pCON2) and two stable plasmids (pCON-S and pCON-2S) derived from the broad-host-range replicon pBBR1 allowing the assessment of plasmid fitness in reciprocal combinations (shown as arrows in the figure). *nptII*, kanamycin resistance gene; *cat*, chloramphenicol resistance gene, *oriV*, origin of replication; *rep*, replication initiation; P*nptII*, native promoter of the *nptII* gene from Tn*5.* Relevant restriction sites are indicated. Previously, we showed that pCON instability is caused by transcription-replication conflicts of the resistance gene transcription and the plasmid replication machinery (Wein et al., 2019). The two stable plasmids (pCON-S and pCON-S2) are characterized by the presence of a longer segment between the resistance gene and the origin of replication in comparison to the unstable plasmids (marked by a triangle).

To study the intracellular dynamics of different plasmid variants, we developed an experimental system that enables us to perform pairwise plasmid competition experiments in *Escherichia coli* K12 MG1655. For the comparison of plasmid fitness between plasmid variants, we performed pairwise plasmid competitions between two variants of the same model plasmid in two settings: in the direct ‘head-to-head’ competition, pairs of plasmids are competing upon arrival into the same naïve host cell. In the preemptive competition, an invading plasmid is competed against an endemic plasmid (i.e., a plasmid that is already present in the host cell) (Figure 1A). Our system includes two variants of the same plasmid backbone: pCON and pCON-S. Both plasmids originated from the pBBR1 backbone isolated from *Bordetella bronchiseptica* (Antoine and Locht, 1992); the pBBR1 plasmid is typically characterized by a small genome size and it is widely spread in diverse environments (Wein et al., 2019). The plasmids pCON and pCON-S are non-mobile, encode the kanamycin resistance gene *nptII* and have a comparable plasmid copy number ranging between 2 and 6 (Wein et al., 2019, 2020). While pCON is characterized by an unstable inheritance in a population, and accordingly is lost over time, pCON-S is characterized by a stable inheritance in the population over long time scales (Wein et al., 2020). The instability of pCON is a result of transcription-replication conflicts of the plasmid replication machinery and the transcription of the *nptII* gene (Wein et al., 2019) that is resolved by an insertion between the *nptII* gene and the *oriV* region in pCON-S (Wein et al., 2020) (see details in Figure 1B) (Wein et al., 2019, 2020). Both pCON and pCON-S have no measurable effect on host fitness (Wein et al., 2019, 2020). To compete both plasmid variants in the same host, we created pCON2 and pCON-S2 in which we replaced the kanamycin resistance gene *nptII* with the *cat* gene that confers resistance to chloramphenicol (Figure 1B). Testing the plasmid stability in an overnight incubation (ca. 8 generations) showed that pCON2 had a loss frequency of 11% ± 3.5 (SE; *n* = 6), which is comparable to pCON, while pCON-S2 had no measurable loss (*n* = 6). Both pCON2 and pCON-S2 had no measurable effect on the host fitness (Supplementary Figure S1). Thus, hosts of all plasmid variants in our experiment had a similar fitness under the tested conditions such that differences in plasmid frequency in the population over time can be attributed to differences in the success of the plasmid to complete a full replication and segregation cycle rather than the fitness of their host.

In the competition experiments, the two plasmid variants were transformed into an *E. coli* population and the presence of both plasmids was validated according to the host resistance to both antibiotics. Hosts of both plasmids were plated on non-selective media and incubated overnight, which amounts to ca. 25 generations. The frequency of both competing plasmids was estimated from the frequency of hosts harboring the plasmids within the population (i.e., as observed in single colonies). Considering the negligible effect of our model plasmids on the host fitness and the minimal number of host cell divisions in the experiment, the observed differences in plasmid frequencies within the population can be interpreted as evidence for differences in the fitness of individual plasmids within the host. Our approach is parallel to the Luria–Delbrück experiment (termed fluctuation test) that was conceived in order to test for a deviation in the frequency of newly emergent chromosomal mutations from the random expectation (Luria and Delbrück, 1943). Using the same logic, if the reproductive success of both competing plasmids is similar then their frequency in the host is expected to be the result of random plasmid segregation only, such that the frequency distribution will be similar between the two plasmids across replicates. Alternatively, if plasmid stability were a determinant of plasmid reproductive success then we would expect the stable plasmid to be significantly more frequent in comparison to the unstable plasmid across replicates.

### The Stability of Plasmid Inheritance Is a Determinant of Plasmid Fitness

**FIGURE 2:**
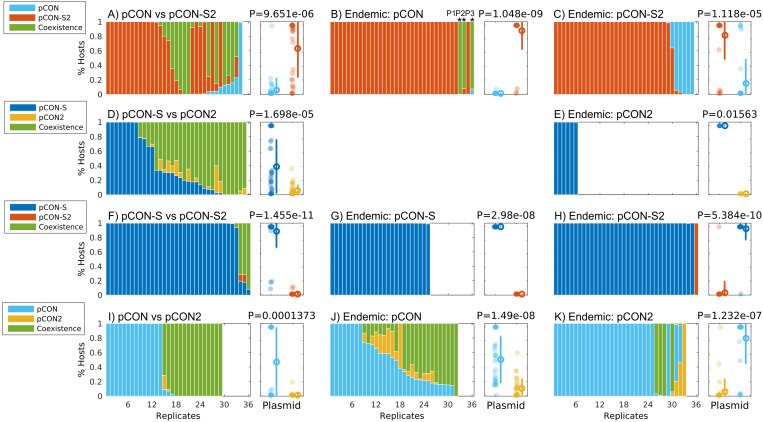
Plasmid competition experiments reveal fitness differences between competing plasmids. Plots **(A–K)** show the results of plasmid competition experiments. The stacked bar graphs show the results of all replicates in the competition experiment with shaded area in the bars proportional to the proportion of host types in the population (see legends). The scatter plots right of each bar graph show a comparison of the proportion of hosts of the two competing plasmids with dot color according to the plasmid type (see legend) and an error bar depicting the mean and standard deviation. The proportion of hosts was compared between the two competing plasmids using a paired sign test (Zar, 2013) with the null hypothesis H_0_: the frequency of the plasmid with the lower mean proportion of hosts (i.e., less frequent) is lower or equal the frequency of the plasmid with the higher mean proportion of hosts (i.e., more frequent). Replicates marked by * in **(B)** were further evolved in a serial transfer experiment (see Figure 4). A comparison of host composition in the evolved populations reveals no clear clustering of replicates derived from the same ancestral clone (Supplementary Figure S3), hence we consider the variability among replicates as independent of their ancestral population. Competitions where the unstable plasmid gained a majority in the population, including those depicted in subplots **(A,H,K)**, were further analyzed by plasmid preparations of sampled colonies. The plasmid content in those replicates validated that, indeed, they were overtaken by the unstable plasmid.

To evaluate the impact of plasmid stability on the reproductive success (i.e., fitness) of competing plasmids, we performed direct competitions between the unstable pCON and the stable pCON-S2 as well as the reciprocal set of pCON2 and pCON-S. Plasmid frequency in the population was measured using the proxy of the proportion of hosts harboring the two plasmids. The results of the head-to-head competition between pCON and pCON-S2 reveal that the frequency of the stable pCON-S2 hosts was higher than that of the unstable pCON hosts (Figure 2A). The stable pCON-S2 was present in most replicate populations (28 out of 35), where the majority of the populations harbored only pCON-S2 hosts hence in these populations pCON went extinct. Overall, pCON was observed in only a few populations (10 out of 35) where pCON hosts typically comprised a minority within the host population. One replicate population stood out as an exception, in which pCON hosts comprised the majority of the population. Additionally, cells hosting a combination of both plasmids in a co-existence were observed in 16 of the populations, where they often comprised the majority of hosts and up to 100% hosts in two of the replicates. The results of the reciprocal head-to-head competition between pCON2 and pCON-S reveal a similar pattern, where the frequency of the stable plasmid pCON-S hosts was higher than that of the unstable pCON2 hosts (Figure 2D). At the end of the competition, the stable plasmid pCON-S was present in most of the replicates (29 out of 36) with eight replicates comprising 100% pCON-S hosts. The unstable plasmid pCON2 was observed in 16 of the replicates, albeit with a low proportion of hosts (<23%). Hosts harboring both plasmids where observed in 27 populations and their frequency was generally high, approaching 100% of hosts in six of the populations (Figure 2D). Both head-to-head competitions show that when an unstable plasmid is in direct competition with a stable plasmid it may be lost from the population. Furthermore, our results show that the loss frequency of pCON while in competition with a stable plasmid is much higher in comparison to the pCON loss frequency observed in a pCON host-only population. Nevertheless, our results reveal a high frequency of cells that host both plasmids, i.e., where the unstable and stable plasmids coexist.

The emergence of segregants (i.e., plasmid-free cells) was observed in both head-to-head competition experiments. The plasmid loss frequency in the pCON/pCON-S2 competition was quite moderate, affecting 13 (36%) of the populations (Supplementary Figure S2). In comparison, plasmid loss frequency in the reciprocal pCON2/pCON-S competition was pervasive, affecting 29 (80,5%) of the populations with a median of 50% segregants per population (Supplementary Figure S2).

Overall, the results of the head-to-head competition reveal a significant deviation from the random expectation as the stable plasmid had a higher frequency in the population in comparison to the unstable plasmid across replicates. Consequently, we conclude that the stable plasmid had a higher fitness in comparison to the unstable plasmid. Our results further show that a competition among plasmids in the cell may lead to the exclusion and extinction of unstable plasmids.

### Plasmid Fitness Depends on the Order of Plasmid Arrival in the Host

The establishment of plasmids in a new host may depend on the presence of other resident plasmids in the host (Kusano et al., 1995; Cooper and Heinemann, 2000). To further test if the effect of plasmid stability on plasmid fitness depends on the plasmid order of arrival, we performed preemptive competition experiments where the competitor plasmid – termed here invading plasmid – is transformed into a host population of the contender plasmid – termed here endemic plasmid. The results of the pCON/pCON-S2 preemptive competitions reveal that the stable plasmid pCON-S2 had a higher frequency in the population in comparison to the unstable plasmid pCON, regardless of order of plasmid arrival, i.e., as the endemic or invading plasmid (Figures 2B,C). When pCON was the endemic plasmid, hosts of pCON-S2 comprised the majority in most replicates while pCON hosts went extinct in most populations. In addition, we observed the emergence of hosts where both plasmids were maintained in coexistence, which comprised ca. 100% of the population in three of the replicates (Figure 2B). When pCON was the invading plasmid, we observed several replicates that included pCON hosts, with five replicates where pCON outcompeted pCON-S2 (Figure 2C). To perform the reciprocal preemptive competition of pCON-S and pCON2 we transformed populations of endemic pCON-S with an invading pCON2, and the other way around. The experiment with pCON-S as the endemic plasmid yielded no transformant colonies, while having pCON2 as the endemic plasmid yielded only a single colony. The overnight pCON2/pCON-S competitions showed that pCON-S hosts comprised the majority of the population, while pCON2 went extinct in all six replicates. Hosts maintaining both plasmids in coexistence were observed with a low frequency (Supplementary Table S2). We note that the pCON2/pCON-S head-to-head competition was characterized by a high proportion of segregants (Supplementary Figure S2), suggesting that the combination of pCON2 and pCON-S cannot be stably maintained within the population.

Taken together, the results of preemptive competition experiments demonstrate that the stable plasmid had a higher fitness in comparison to the unstable plasmid, regardless the order of arrival and the plasmid marker genes *nptII* and *cat*.

### Plasmid Fitness Advantage Depends on a Plasmid Encoded Trait

Our results so far show that plasmid stability determines plasmid fitness. Nonetheless, plasmids may differ not only with regards to their stability, but also in the traits that they encode. To further examine the effect of plasmid-encoded traits on the plasmid fitness, we compared the fitness of plasmids having similar stability but differ in the antibiotic resistance gene that they carry. For that purpose, we performed pairwise competition experiments between the two stable plasmid variants and between the two unstable plasmid variants.

The results of the head-to-head competition between pCON-S and pCON-S2 showed that the frequency of pCON-S hosts was higher than that of pCON-S2 hosts, which were observed in only three replicates in a very low frequency (Figure 2F and Supplementary Table S2). In addition, we observed the emergence of plasmid coexistence in several replicates with hosts of both plasmids comprising the majority of the population (>60%) only in three replicates (Figure 2F). The preemptive competitions performed with both stable plasmids show that pCON-S hosts comprised the majority of replicates regardless of whether pCON-S was the endemic or the invading plasmid (Figures 2G,H). The reciprocal preemptive competition with pCON-S2 as the endemic plasmid resulted in similar dynamics with the majority of replicates (31 out of 36) and one exceptional replicate where only pCON-S2 hosts where observed (Figure 2H and Supplementary Table S2). Plasmid loss in all competition experiments was minimal when pCON-S was the endemic plasmid and slightly higher when pCON-S2 was the endemic plasmid (Supplementary Figure S2).

Comparing the proportion of pCON-S and pCON-S2 hosts in all competition experiments shows that pCON-S maintained a higher frequency in the population regardless of the order of arrival. The results of the competition experiments between stable plasmids thus show that the presence (or invasion) of a stable plasmid may lead to the exclusion of an equally stable plasmid type. We observe a clear fitness advantage of the *nptII*-encoding plasmid over the *cat*-encoding plasmid despite their equal stability. Whether the expression of *nptII* or *cat* has an impact on the host fitness under the conditions we applied in our experiments remain unknown. Our results thus indicate that in a competition between equally stable plasmids, other plasmid properties may be important determinants of the plasmid fitness.

The results of the head-to-head competition of both unstable plasmids – pCON and pCON2 – show that half of the replicates included a majority of pCON hosts, while the other half included a majority of host where both plasmids coexisted (Figure 2I). The presence of pCON2 could be observed only in two of the replicates, albeit, at a very small frequency. A similar trend was observed in the results of the preemptive competitions between the two unstable plasmids. When pCON was the endemic plasmid and pCON2 the invading plasmid, the frequency of pCON hosts was higher than the frequency of pCON2 hosts (Figure 2J). The presence of the invading plasmid pCON2 was observed in 19 of the replicates, with a maximum frequency of 60% (Supplementary Table S2). The presence of pCON and pCON2 was observed in most replicates, with hosts of both plasmids in coexistence making up for the majority in 14 (44%) of the replicates. In the reciprocal preemptive competition with pCON2 as the endemic plasmid and pCON as the invading plasmid, the frequency of pCON hosts was higher in comparison to the frequency of pCON2 hosts (Figure 2K). The presence of pCON2 could be observed only in five replicates with one replicate including only pCON2 hosts. Additionally, we observed the emergence of hosts of both plasmids in coexistence in seven replicates, with four replicates comprising mostly hosts of both plasmids (Figure 2K and Supplementary Table S2). The plasmid loss frequency was similar among the three pCON/pCON2 competitions (Supplementary Figure S2). The results of the competition experiments between the two unstable plasmids show that pCON had a fitness advantage over pCON2. The results of the pairwise competition experiments between the stable or unstable plasmid pairs show that the same plasmid type (i.e., encoding the same trait) had a fitness advantage over the other plasmid type regardless of plasmid stability.

### Competing Plasmids Are Maintained via Plasmid Fusion Over Time

**FIGURE 3:**
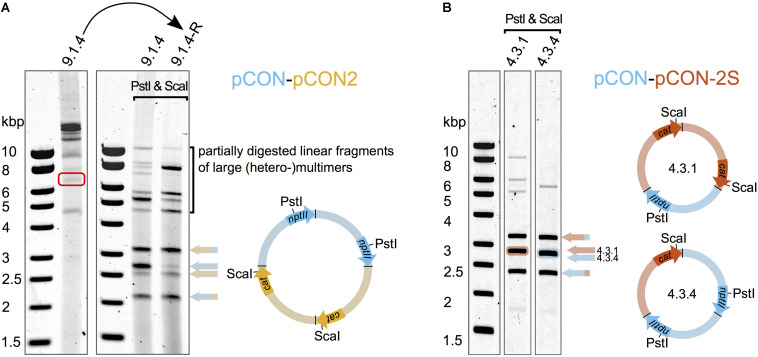
Plasmid fusions produce inheritable heteromultimeric plasmids. Heteromultimer specific restriction products were obtained after digesting the plasmid DNA with PstI and ScaI that cleave as indicated in the schematic plasmid maps. The specific products are indicated by colored arrows whose pattern match the pattern of the respective restriction fragment in the plasmid maps. The heteromultimeric plasmids are depicted as multimers with colors corresponding to the competed plasmids (Figure 1). The head to tail orientation of the individual plasmid units is indicated by arrows of the respective marker genes *nptII* and *cat* (according to plasmid directionality shown in Figure 1). **(A)** Diverse plasmid (hetero-)multimers dominate the total plasmid content at the end of the competition. A representative clone (9.1.4) from the “head-to-head” competition between the unstable plasmids pCON and pCON2 was analyzed to test whether hetero-multimeric plasmid form is inheritable. A heteromultimer was isolated from a non-treated DNA preparation (red rectangle) and used for the re-introduction into *E. coli* MG1655 (curved arrow). **(B)** Plasmid heteromultimers can be stably maintained. Plasmid content analysis of representative clones from the preemptive competition experiment between the stable plasmid pCON-2S and the invading unstable plasmid pCON at the end of the serial transfer experiment shown in Supplementary Figure S4. Note that the plasmid content shown in **(A)** is comprised of various multimer types and not dominated by a specific type of heteromultimer as shown in **(B)**. The full plasmid content analysis with an extended explanation is shown in Supplementary Figure S4. Our results further show that pBBR1-like plasmids may recombine to form fusion plasmids (plasmid multimers) that can replicate in presence of multiple origins of replication (*oriVs*).

Our results show that different plasmid variants can be maintained in coexistence. Plasmid coexistence may be explained either by plasmid co-residence in the cell or by plasmid recombination (i.e., plasmid fusions). To further investigate the genetic basis of plasmid coexistence, we analyzed the plasmids from double resistant colonies from each of the “head-to-head” competition experiments. In all of the plasmid hosts we observed large plasmids much above the size of our model plasmids (up to ∼20 Kb). Restriction enzyme digestion analysis showed that these large plasmids corresponded to fusions of both competing plasmids. Hence, the plasmids recombined to form heteromultimeric structures (Figure 3A). We did not observe any apparent re-arrangements or deletions in the plasmid genomes; instead the multimers were composed of the intact plasmids in tandem repeat orientation (Figure 3A). Notably, different heteromultimeric combinations appeared in variable frequencies across plasmid hosts such that no two plasmid populations looked alike. The presence of such diverse combinations of multimers in single hosts suggests that the formation and diversification of plasmid multimers is a dynamic and rapid process. In order to assess whether the observed multimeric plasmids are heritable, we excised a plasmid multimer from an agarose gel and transformed it into an *E. coli* MG1655 host. Indeed, we obtained a double resistant clone and subsequent DNA analysis validated that the excised plasmid contained a heteromultimeric plasmid fusion. A diversity of plasmid fusions was reconstituted already after overnight incubation, adding further support that the process of plasmid diversification is rapid. Our results thus demonstrate that plasmid coexistence is achieved by plasmid fusions that are heritable.

**FIGURE 4:**
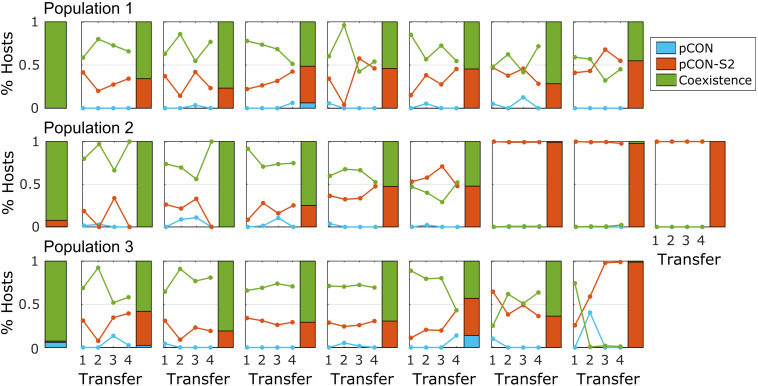
Plasmid coexistence is stable over time. Each subplot shows the results of a short-term evolutionary experiment for a single replicate population with rows corresponding to the three ancestral populations. The solitary stacked bars on the left show the initial host composition in the ancestral population (as in Figure 2). Lines in the plots show the proportion of hosts over the experiment. The host composition at the end of the experiment is shown by a stacked bar. The frequency of segregants (i.e., plasmid loss) reached 100% in two of the replicates; otherwise, plasmid loss was rather low with a median of 1.4% segregants per day in the total replicates. The full data set is provided in Supplementary Table S3.

To test the persistence of such plasmid fusions in the population over time, we performed a short-term evolution experiment of representative populations where hosts of both plasmids comprised the majority of the population. For that purpose, we selected three populations from the pCON/pCON-S2 competition experiment (Figure 2B). Eight replicates from each population were evolved in serial transfer for 4 days, that is, ca. 80 generations. The experiment results reveal that hosts of both plasmids in coexistence were maintained in the population over time in most replicates (Figure 4). The fixation of plasmid coexistence (i.e., >90% of hosts) was observed in two replicates, while in the other replicates we observed the emergence of separate populations of pCON and pCON-S2 hosts. In four of the replicates, hosts of pCON-S2 constituted the majority of the population at the end of the experiment. Our results thus indicate that plasmid coexistence is a reversible state where both stable and unstable plasmids are able to segregate from hosts carrying plasmid fusions.

To investigate the genetics of the plasmid coexistence in the evolved populations, we analyzed plasmid DNA of representative hosts from eight of the evolved populations (4.1 and 4.3). Again, we observed heteromeric plasmid fusions in all of the analyzed samples, albeit at a much reduced diversity (Figure 3B). The decreased diversity of plasmid multimers allowed us to resolve some of the plasmid fusions into distinct plasmid compositions. Examples of heteromultimeric structures include oligomers where two pCON plasmid molecules were fused with one molecule of plasmid pCON-S2, forming a heterotrimer (Figure 3B). Notably, we frequently observed faint bands that matched the size of supercoiled monomers of the model plasmids, suggesting that the plasmid coexistence state is either accompanied by the persistence of plasmid monomers or their reappearance through intramolecular recombination within multimers (Supplementary Figure S4B). This observation is further supported by the segregation into plasmid-specific hosts we observed in the serial transfer experiment (Figure 4). Our results thus show that the competing plasmids can be maintained in coexistence following plasmid fusions that can maintain the unstable plasmid over time. Furthermore, we show that plasmid fusion diversity is reduced over time, which indicates that certain plasmid fusions may have a fitness advantage over others.

## Discussion

Stable reproduction and segregation during cell division are essential for plasmid persistence in the population over time. Increasing evidence shows that the persistence of mobile plasmids under strong selection for the plasmid-encoded function is mainly governed by vertical inheritance, while lower selection pressure for the plasmid leads to persistence by mobility in the population (e.g., Lopatkin et al., 2016; Hall et al., 2017; Stevenson et al., 2017). Strong selective conditions for the plasmid presence eliminate non-hosts from the population, thus revealing plasmids that maintain (or adapt) a stable inheritance with the host. When the strength of selection is reduced (or eliminated), plasmids whose segregation is incomplete can persist in the population by alternative mechanisms [e.g., mobility (Hall et al., 2016, 2017; Gumpert et al., 2017; Lopatkin et al., 2017)]. Using our approach for direct *in cellulo* plasmid-competitions, we demonstrate the importance of plasmid stability for the evolutionary success (i.e., fitness) of plasmids, even under very short time scales. Nevertheless, our results reveal that unstable plasmid variants may, in several cases, rise to a high frequency in the population (e.g., Figure 2C). Previously we suggested that the effect of selection on plasmid alleles is reduced due to random genetic drift of plasmid alleles during cell division – termed segregational drift (Ilhan et al., 2019). Since the dynamics of plasmid alleles under non-selective conditions largely depends on allele frequency in the population (Ilhan et al., 2019), a stable plasmid may still be lost from the population when its initial frequency in the ancestral cell is low. Our results thus support the notion that segregational drift of plasmid alleles may lead, in some cases, to the fixation of inferior plasmid variants in the population.

Plasmid persistence in a host may depend on an interaction with co-residing plasmids (e.g., San Millan et al., 2009, 2014; Silva et al., 2011; Dionisio et al., 2019; Jordt et al., 2020) or on their recombination and fusion with other plasmids. Recombination routes were shown to facilitate the formation of mosaic plasmids, a phenomenon often observed in different plasmids of the same host lineage (e.g., Chaconas and Norris, 2013; Pesesky et al., 2019). Recombination observed between sites in a single plasmid genome may indicate that plasmid recombinants form routinely when host recombination pathways are encoded in the host genome (e.g., Flores et al., 2000). Indeed, plasmid recombination is dependent on the host recombination pathways and the formation of plasmid oligomers occurs along the normal lifecycle of plasmids (e.g., James et al., 1982; Chédin et al., 1997). The main cause for the formation of dimers and higher multimers is homologous recombination between spatially linked sister plasmids during or after replication. In contrast, the formation of heteromeric plasmids depends on the recombination of non-sister plasmid molecules (i.e., recombination between two different plasmids). When recombination between non-sister molecules happens early after entry of an invading plasmid (or two plasmids invading at the same time), recombination could lower the effective plasmid copy number of the individual plasmids available for segregation into daughter cells and act as purifying selection toward the existence of the two competing plasmids in a hybrid state (i.e., a heteromer) under double selection. In the competition experiments involving the unstable plasmid pCON2, where we observed higher frequencies of hosts maintaining both plasmids in coexistence (Figures 2D,I,J), such early recombination events might have happened, thereby allowing pCON2 to escape from rapid extinction. Since our experimental design required one step of double selection after plasmid delivery into the host cells, such heteromultimeric forms might have had higher fitness than the individual monomers and were more rapidly purified.

Since the competing plasmid pairs in our experiments share full sequence identity, except for the marker genes *nptII* and *cat*, the occurrence of rearrangements within the heteromeric plasmids is expected (Doherty et al., 1983). However, we did not observe any apparent rearrangements in the analyzed heteromultimeric plasmids; all of them comprised units of the model plasmids in tandem orientation. It is likely that the non-identical gene sequences between the plasmid units in a heteromultimer limited recombination to single crossover events, which led to either demultimerization or the formation of higher multimers (i.e., in the recombination between different plasmid molecules). Indeed, the resolution of dimers and higher order multimers back to monomeric forms is possible via intramolecular homologous recombination (Kolodner, 1980) or through site-specific recombination that is mediated by a plasmid – or host – encoded multimer resolution system (e.g., Stark et al., 1992; Fournes et al., 2016). Consequently, the observed stability of heteromultimeric plasmid fusions in our experiments is most likely the result of slow multimer-to-monomer conversion rates by the host-encoded recombination pathways (Summers et al., 1993) and the lack of an apparent multimer resolution site in our model plasmids.

In our experiment, a short-term selective event of both antibiotics was sufficient to select for the larger fusion plasmids. Our results reveal a diversification process of plasmid fusion variants over time that leads to the enrichment of selected variants. Selection for antibiotics resistance may preserve unstable plasmid variants in the population (Wein et al., 2020); our results here show that such unstable plasmids can be maintained in the population over time also under non-selective conditions via plasmid fusion. Plasmid fusion of plasmid alleles conferring resistance to different antibiotics has been invoked as a likely route for the evolution of plasmids carrying multiple antibiotics resistance genes (Condit and Levin, 1990). Indeed, the emergence of plasmids conferring resistance to different antibiotics is conceivable, e.g., for plasmids having a high copy number and hence an increased mutational supply (Rodriguez-Beltran et al., 2018), or following gene acquisition by lateral gene transfer [i.e., homologous recombination (Norberg et al., 2011)]. The results of our experiment suggest that once plasmid fusions are formed – in our experiment due to a single selection event – they can enable a stable inheritance of resistance to multiple antibiotics over time also under non-selective conditions. Thus, plasmid stability that is facilitated by plasmid fusion may be a route of plasmid long-term persistence, especially in environments with fluctuating selection pressure.

The comparison of plasmid evolutionary success demonstrates that plasmids variants may differ in their fitness. Our results support the notion that natural selection operates on plasmids in two hierarchical levels: one component is the plasmid fitness within the cell, and the other is the host fitness within the population. Notably, similar principles may apply to the evolution of other extra-chromosomal elements such as mitochondria (Taylor et al., 2002; Gitschlag et al., 2016) and viruses (Krakauer and Komarova, 2003). Our results thus suggest that plasmids in a host cell should be viewed as Darwinian entities whose evolution is governed by basic principles of population genetics, a process partially independent of the hosting bacterial populations. Plasmids are not only useful accessory genetic elements to their host but they are also evolving and replicating entities, similarly to cellular living forms. There is a clear link between plasmid genetics and plasmid evolutionary success – hence plasmids are evolving entities whose fitness can be quantified.

## Materials and Methods

### Bacterial Strains and Culture Conditions

The wild-type laboratory *E. coli* K-12 strain MG1655 was used as the model organism (DSM No. 18039, German Collection of Microorganisms and Cell Cultures, DSMZ). *E. coli* DH5α (Hanahan, 1983) was used during plasmid constructions and for the analysis of re-introduced plasmids from the plasmid fitness assays (see below). All strains were routinely grown at 37°C in lysogeny broth (LB) medium at 250 rpm shaking or on LB-agar plates. Antibiotics for the selection of plasmid carrying host cells were used at the following concentrations: kanamycin 25 μg/ml (pCON and pCON-S hosts), chloramphenicol 10 μg/ml (pCON2 and pCON-S2 hosts), kanamycin 25 μg/ml and chloramphenicol 10 μg/ml (double-plasmid hosts carrying one of the four different possible plasmid combinations).

### Plasmid Constructions

All plasmids in this study were constructed using the Gibson assembly technique (NEBuilder^®^ protocol; New England Biolabs). All primers are listed in Supplementary Table S1. Plasmid pCON-S was derived from plasmid pCON (GenBank accession no. MK697350) by insertion of a 254 bp PCR product covering nucleotides 817 to 1044 in pBBR1MCS-5 (GenBank accession no. U25061) into pCON immediately upstream of *oriV* (Wein et al., 2020). Plasmid pCON2 and pCON2-S were constructed by replacing the open reading frame (ORF) of *nptII* in pCON and pCON-S with the ORF of the chloramphenicol resistance-encoding gene *cat*, while keeping the original promoter of the *nptII* gene unaltered. The ORF of the *cat* gene, corresponding to the nucleotides 1 to 660 of the *cat* gene ORF from transposon Tn*9* (GenBank accession no. V00622.1.), was PCR-amplified from plasmid pPCR-Script Cam SK(+) (Agilent technologies), using the primer pair cat-GA-fw and cat-GA-rv. The *nptII*-less backbone of pCON-S was PCR-amplified using the primer pair pCONSinvFw and pCONSinvRv. Plasmid pCON2 was constructed by replacing the ORF of the *nptII* gene in pCON with the ORF of the *cat* gene (see above). Primers used for the PCR amplification of the *cat* ORF were cat-pCON2-GA-fw and cat-pCON2-GA-rv. The *nptII*-less backbone of pCON was PCR-amplified using the primer pair pCON2-GA-fw and pCON2-GA-rv.

### Plasmid Fitness Competition Experiments

The success of two plasmid types competed in either the ‘head-to-head’ or preemptive competition mode was determined in single colonies that had grown up non-selectively during 24 h of growth from single cells that initially carried two plasmids. In order to select for double plasmid carriage and to distinguish between the two plasmids, the competing plasmid pairs harbored different marker genes: *nptII* in the unstable plasmid pCON and the stable plasmid pCON-S and *cat* in the unstable plasmid pCON2 and stable plasmid pCON-S2. ‘Head-to-head’ competitions experiments were initiated by transforming *E. coli* MG1655 with a mixture of two plasmids at the same time (i.e., pCON *and* pCON2, pCON *and* pCON-S2, pCON-S *and* pCON2, and pCON-S and pCON-S2, respectively), giving in total four sets of competition experiments. Likewise, for each preemptive competition experiment *E. coli* MG1655 already carrying one of our four model plasmids was transformed with a compatible plasmid in terms of plasmid-encoded marker gene, resulting in three sets of plasmid-plasmid combinations that were reciprocally performed (i.e., in total six reciprocal plasmid-plasmid combinations). The four combinations were as follows (endemic versus invading plasmid): pCON versus pCON2, pCON versus pCON-S2, pCON-S versus pCON2, and pCON-S versus pCON-S2. All plasmids were introduced in *E. coli* MG1655 by electroporation (1 ng per plasmid DNA per electroporation). To increase the transformation efficiency for double transformations (head-to-head competition experiments) concentrations of 4–10 ng per plasmid DNA were used. Electro-competent cells were prepared as reported (Dower et al., 1988) and plasmid DNA preparations used for electroporation were isolated from *E. coli* MG1655 host cells. Colonies resistant to both kanamycin (*nptII*^+^) and chloramphenicol (*cat*^+^) were selected overnight on selective LB plates. Every competition experiment was initiated with six randomly chosen primary double resistant colonies obtained. The six primary transformants were picked and streaked for single colonies on non-selective LB plates. After overnight incubation, for each of the six primary transformants, six single colonies were excised (*n* = 36 replicates per competition experiment). The colonies were resuspended in 1 ml PBS, serially diluted and aliquots plated on non-selective plates [yielding the total cell number per colony (*N*)] and plated on kanamycin-supplemented LB plates, chloramphenicol-supplemented plates and plates supplemented with both antibiotics (giving the frequencies of cells being *nptII*^+^, *cat*^+^, and *nptII*^+^*cat*^+^). The proportion of cells carrying only one of the two competing plasmids within one colony were calculated as *P*_*p*1_ = (*N*_*nptII*_^+^ - *N*_*nptII*_^+^_*cat*_^+^)/*N* for cells carrying either pCON or pCON-S and *P*_*p*2_ = (*N*_*cat*_^+^ - *N*_*nptII*_^+^*_*cat*_*^+^)/*N* for cells for cells carrying either pCON2 or pCON-S2. The proportion of cells carrying both plasmids was calculated as *P*_*p*1&2_ = *N*_*nptII*_^+^*_*cat*_*^+^/*N*. Plasmid loss was calculated as *P_*loss*_* = 1 - *P*_*p*1_ - *P*_*p*2_ - *P*_*p*1+2_. Our evaluation regime included counting of preferably large and therefore reliable sample sizes for the determination of the different plasmid-carrying cell types. In cases when, due to variability in the plating, colony counts on double selective plates were slightly higher than on plates containing only one of the two antibiotics, counts were corrected toward the counting result on double selective medium in order to avoid negative numerical values in the calculation of the proportions.

### Serial Transfer Experiment/Short Term Evolution Experiment

The evolution experiment was conducted under non-selective conditions and was founded with double resistant colonies from the preemptive plasmid fitness competition experiment between unstable pCON and stable pCON-S2. In three of the replicates of this experiment (i.e., replicates 4.1, 4.3, and 4.5) double resistant cells formed the vast majority in the analyzed colonies at the end of the competition. Because of this dominance, double resistant colonies could be readily picked from non-selective plates that were used to estimate the total number of cells within a colony at the end of the competition. For each the replicates, eight colonies were randomly picked and inoculated into 1 ml LB medium for overnight growth at 37°C with constant shaking. At the onset of the experiment samples were plated on double selective media to confirm the double resistant phenotype of the colonies. The overnight cultures were then serially propagated every 24 h in a volume of 1 ml LB medium for three additional transfers using a 1:1000 dilution factor. In total, we recorded ∼64 generations (∼28 generations for the first growth passage on solid medium and ∼9 generations per transfer in liquid medium). Every day, the proportion of plasmid types in the populations was estimated from the proportion of host cells carrying either pCON or pCON-S2 or both as described above for the plasmid competition experiments. At the end of the experiment an individual double resistant colony from four evolved populations of replicate 4.1 and replicate 4.3 were randomly chosen for plasmid isolation followed by diagnostic restriction enzyme digestion.

### Plasmid Stability Assay

The plasmid loss frequency was estimated from the frequency of plasmid free cells occurring during overnight growth in non-selective media. To determine the loss frequency, cultures were inoculated from single colonies grown on selective media to ensure plasmid carriage. After 12 h growth in 37°C (approximately 8.5 generations), the cultures were serially diluted and plated on non-selective LB media. After overnight incubation, 100 colonies were picked and streaked on selective media (chloramphenicol 20 μg/ml). Colonies grown on selective plates were counted as plasmid carrying. The loss frequency was calculated from plasmid-free cells (not resistant) and the total number of colonies tested.

### Fitness Experiments

The relative fitness (*w*) (Arjan et al., 1999) of the plasmid-carrying versus the ancestral plasmid-free strain (wt) was estimated by pairwise competition experiments. All competition experiments were initiated with a 1:1 mixture of 1:100 diluted plasmid-carrying strain and ancestral strain [*E. coli* MG1655 Tm^*r*^; (Wein et al., 2019)] from overnight cultures, in a total volume of 1 ml of non-selective LB medium. The relative fitness of the plasmid host strains was calculated by evaluating cell counts at the time points 0 and 24 h. The strains were distinguished through plating on non-selective (LB) and selective media (LB supplemented with trimethoprim 150 μg/ml). The chromosomal integration (Tm^*r*^) as well as the plasmid pCON have no measurable impact on the fitness of *E. coli* MG1655 [previously shown in (Wein et al., 2019)].

### Analysis of Plasmids by Restriction Enzyme Digestion

Plasmids were extracted from stationary overnight cultures inoculated from single colonies and grown without selection using the GeneJET Plasmid Miniprep Kit (Thermo Fisher Scientific). Plasmid DNA was quantified using the Multiskan GO spectrophotometer instrument (Thermo Fisher Scientific). Plasmid conformations were analyzed by restriction enzyme digestions using PstI, ScaI or HindIII following the manufacturers’ guidelines (New England Biolabs). Analytical digestions were electrophoresed against a known standard (Hyperladder^TM^ 1 Kb, Bioline) in 1 × TBE buffer at 4.3 V/cm in 0.7% (w/v) agarose gels containing 1 μg/ml ethidium bromide. Gels were destained with deionized water and documented using the ChemiDoc imaging system (Bio-Rad).

### Statistical Analysis

Statistical tests and data analysis were performed in R version 3.5.3 (R Core Team, 2020) and MatLab^©^ version R2015b.

## Data Availability Statement

All datasets generated for this study are included in the article/Supplementary Material.

## Author Contributions

NH, TW, and TD designed the experiments. NH, TW, JE, and AG established and performed the experimental work and analyzed the data. TD supported the data analysis. All authors interpreted the results and wrote the manuscript.

## Conflict of Interest

The authors declare that the research was conducted in the absence of any commercial or financial relationships that could be construed as a potential conflict of interest.
