# Supplementary material for: Intracellular Competitions Reveal Determinants of Plasmid Evolutionary Success
Source: Front Microbiol. 2020 Sep 4;11:2062. doi: 10.3389/fmicb.2020.02062 (PMC7500096; doi:10.3389/fmicb.2020.02062)
Supplement: Supplementary file 1 [file Data_Sheet_1.pdf]

Supplementary Material

Intracellular competitions reveal determinants of plasmid evolutionary success

Nils F. Hülter[1], Tanita Wein[1], Johannes Effe[1], Ana Garoña[1], Tal Dagan[1]

[1]Institute of Microbiology, Kiel University, 24118 Kiel, Germany

* Correspondence:
Tal Dagan
tdagan@ifam.uni-kiel.de

Supplementary Table S1. Oligonucleotides used in this study

**Name**	**DNA sequence (5' to 3')**
**pCON-S2 assembly**	
cat-GA-fw	AGGATGAGGATCGTTTCGC**ATG**GAGAAAAAAATCACTGGATATACCACC
cat-GA-rv	CGTCGAGCCGGTTGGACACC**TTA**CGCCCCGCCCTGC
pCONSinvFw	AGTGGCAGGGCGGGGCGTAAGGTGTCCAACCGGCTCG
pCONSinvRv	CCAGTGATTTTTTTCTCCATGCGAAACGATCCTCATCCT
**pCON2 assembly**	
cat-pCON2-GA-fw	CAGGATGAGGATCGTTTCGC**ATG**GAGAAAAAA
cat-pCON2-GA-rv	CGAGCCAGCCGGTGGCCGCT**TTA**CGCCCCGCCCTGC
pCON2-GA-fw	AGTGGCAGGGCGGGGCGTAAAGCGGCCACCGG
pCON2-GA-rv	CCAGTGATTTTTTTCTCCATGCGAAACGATCCTCATCCT

Overhangs that generated sequence identity to the target fragment in the respective assembly reaction are underlined. Translation start and stop sites are shown in bold typeface.

Supplementary Table S2. Proportion of host types in the competition experiments (data depicted in Figure 2). See csv file.

Supplementary Table S3. Proportion of host types in the serial transfer experiment (data depicted in Figure 4). See csv file.

Figure S1. Plasmid stability and relative fitness of plasmids pCON2 and pCONS2. **A**, Plasmid loss after overnight incubation. **B**, Relative fitness of plasmid carrying strain after competition against marked wildtype strain. Each experiment was carried out with n=6 biological replicates. The mean fitness effect observed for pCON2 was 0.98±0.024($CI_{95\%}$) and for pCON-S2 1.006±0.031 ($CI_{95\%}$). Since the confidence interval of the mean fitness effect of both plasmids includes *w*=1 we conclude that no fitness effect could be observed for both plasmids.

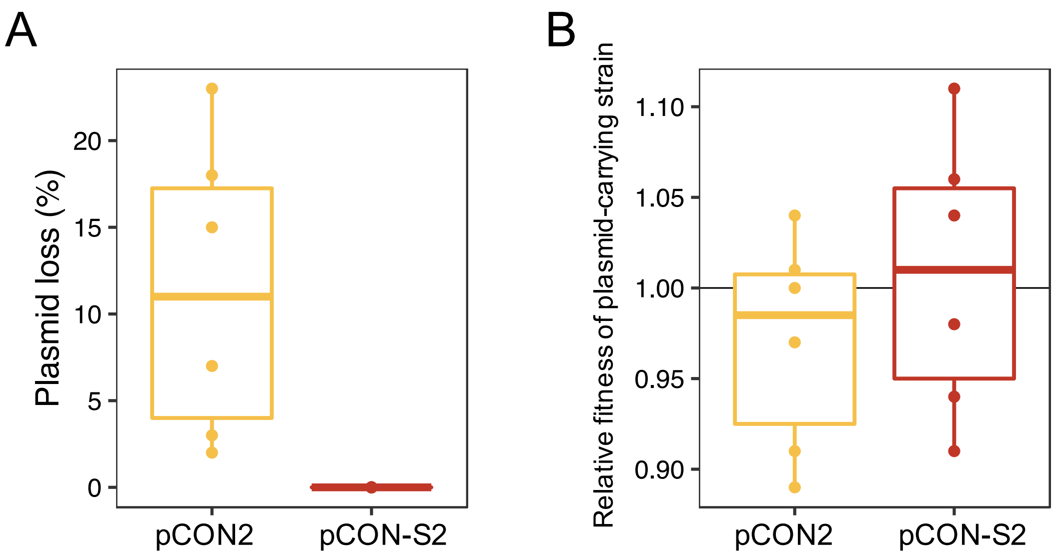

Figure S2. Frequency of plasmid loss during the competition experiments. A cumulative distribution function (CDF) of the proportion of segregants in all replicates for each competition experiment is shown. Competitions of plasmid pairs are grouped in the same plot. Note that since the experiments were performed on solid media, plasmid loss may occur already during the incubation on selective media due to the colony structure (e.g., Gralka and Hallatschek, 2019).

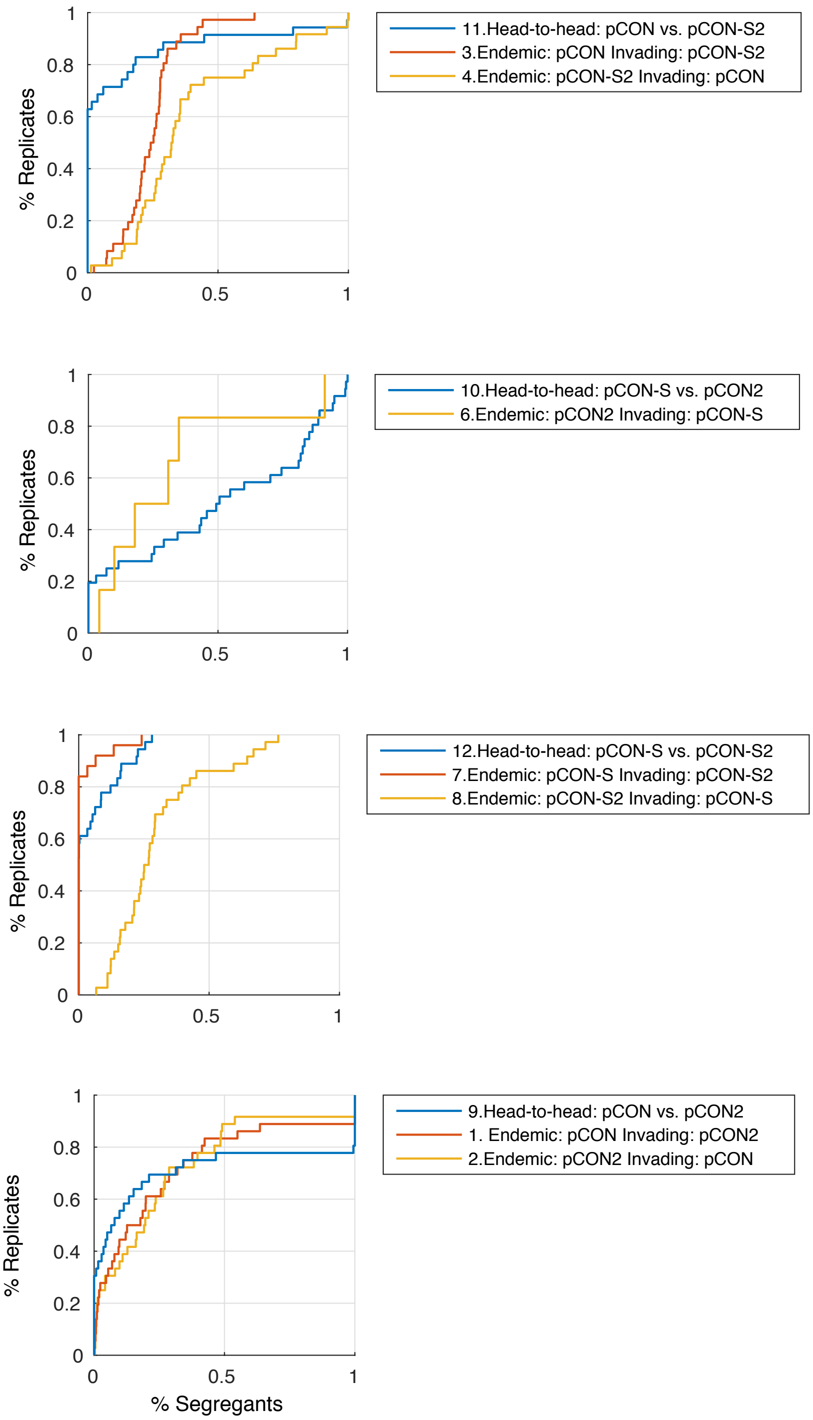

Figure S3. Variability among replicates originating from the same ancestral clone visualized as a principle component analysis (PCA). Each plot corresponds to replicates in a competition experiment as in Fig. 2. Variables in the PCA include the proportion of different host types (plasmid specific or coexistence) and segregants; their trajectory is shown by a blue line. The first two components are shown (termed PC1 and PC2). Dots symbols correspond to replicate populations with are colored according to their ancestral clone (up to six clones; see methods for details).

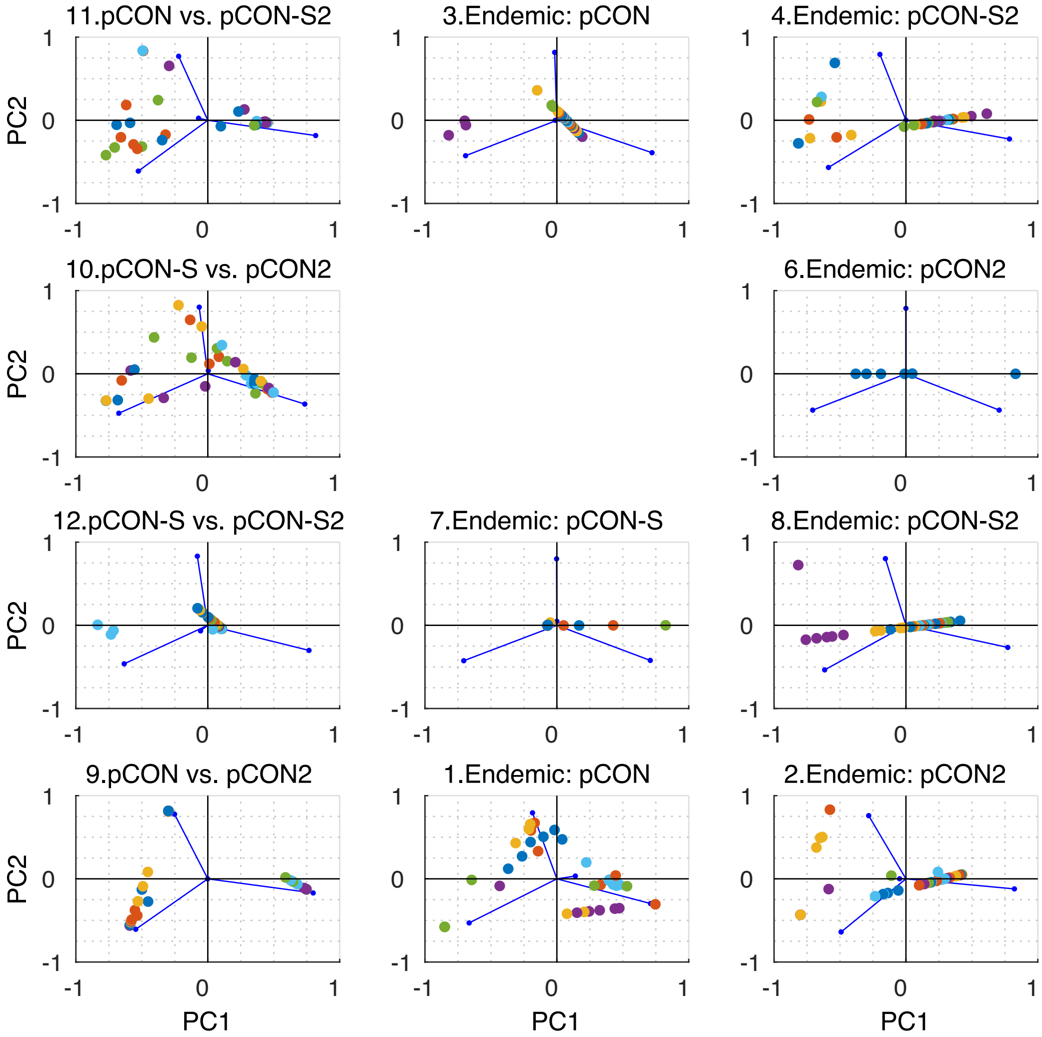

Figure S4: Plasmid DNA content of clones carrying heteromultimeric plasmids analyzed by restriction digestion analysis and one-dimensional agarose gel electrophoresis; treatments are indicated above the respective lanes (-, untreated; B, Nb.BsrDI; H, HindIII; P, PstI; S, ScaI; P+S, double digestion with PstI and ScaI). **A:** Diverse plasmid fusions formed during the “head-to-head” plasmid competition between pCON and pCON2. Lanes 1 to 5 and lane 11: Plasmid DNA isolated at the end of the competition experiment from one representative clone (clone 9.1.4). Lanes 6 to 10 and 12: Plasmid DNA isolated from one double resistant transformant (9.1.4-R) that was obtained after transformation of *E. coli* MG1655 with a circular heteromultimer (see red rectangle in lane 1). DNA bands of relaxed circular plasmids obtained after nicking with Nb.BsrDI indicate the existence of several large plasmid multimers (lanes 2 and 7). Digestion with HindIII, which cuts once in pCON and pCON2, produces equal proportions of linear plasmid monomers (lanes 3 and 8). Digestion with PstI, which cleaves in *nptII* gene of pCON, results in the release of linear pCON monomers that can only occur when two copies of pCON form direct tandem repeat in a heteromultimer (lanes 4 and 9). ScaI, which cleaves in the *cat* sequence of pCON2, creates a similar pattern as observed with PstI. The presence of large linear fragments in both digestions indicates that the plasmids DNA content is not comprised of homomultimers of both plasmids, but of heteromultimers with different composition. Double digestion with PstI and ScaI provides proof for the presence of heteromultimeric plasmids. **B:** Plasmid DNA content analysis of two representative double resistant clones from the competition between pCON and pCON-S2 (pre-emptive mode) isolated at the end of the short-term evolutionary experiment presented in Figure 4. Plasmids from hosts carrying either pCON or pCON-2S were included for direct comparability (lanes 1 to 4). Note the presence of faint bands that match the size of supercoiled monomers of the model plasmids (lanes 7 and 10). Double digestion with PstI and ScaI provides proof for the presence of heteromultimeric plasmids in both clones (lanes 9 and 14). Treatment with HindIII produces linear plasmid monomers (lanes 6 and 11). Note that due to the small size difference between pCON and pCON-2S, the two linear restriction products did not resolve into separate bands. The plasmid DNA content of clone 4.3.1 is dominated by a heterotrimer comprised of one copy of pCON and two copies of pCON-2S. In this constellation, PstI cleaves only once in the *nptII* gene (lane 7), whereas ScaI cleaves two times, producing one linear monomer of pCON-2S (lane 8). The plasmid DNA content of clone 4.3.4 is dominated by a heteromultimer in which two copies of pCON and one copy of pCON-2S created a hetetrotrimer. In this constellation PstI cleaves twice (lane 12), whereas ScaI cleaves only once (lane 13).

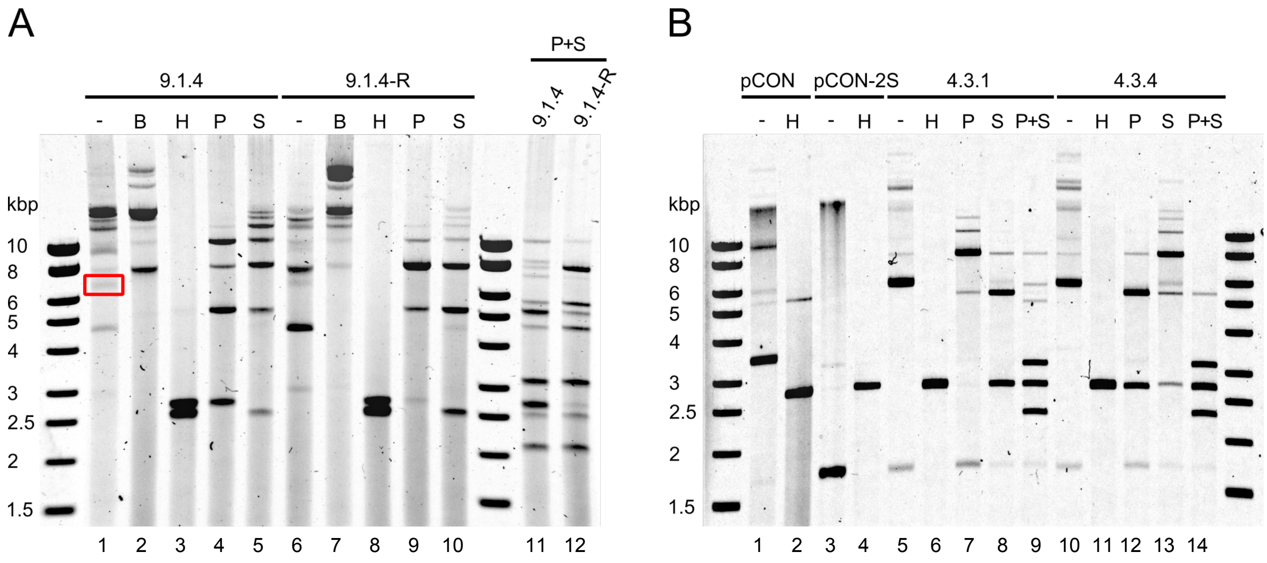

Supplementary methods for Figure S4: For the transformation experiment described above in Figure S4A, plasmid DNA was isolated from one double resistant colony (9.1.4) that was obtained in the “head-to-head” competition experiment between pCON and pCON2. About 50 ng of untreated plasmid DNA was subjected to agarose gel electrophoresis using a 0.7% gel and TAE buffer. After staining with Midori Green, one band (as indicated above in Fig. S4A) was identified as closed circular DNA that matched the expected size of a heteromultimeric plasmid. The band was excised from the gel and the DNA extracted using the freeze and squeeze extraction principle. The DNA was used for transformation of *E. coli* MG1655 and double resistant transformant colonies were selected on LB plates supplemented with kanamycin (25 µg/ml) and chloramphenicol (10 µg/ml). One double resistant colony was chosen (9.1.4-R), and re-streaked on double selective medium. For the plasmid isolation, cells grown overnight were scraped off the agar surface and used for plasmid isolation using the extracted using the GeneJET Plasmid Miniprep Kit (Thermo Fisher Scientific). Plasmid DNA content analysis was carried out using Plasmid-Safe ATP-dependent Exonuclease (Epicentre) for the removal of linear DNA from the plasmid preparation prior to all other enzymatic treatments. The enzymes Nb.BsrDI, HindIII, PstI, and ScaI (all NEB Biolabs) were used for analytical restriction digestions as recommended by the manufacturer. Note the plasmid preparations shown in Figure S4B were not treated with Plasmid-Safe ATP-dependent DNase prior to the restriction digestion analysis.

Reference

Gralka, M., and Hallatschek, O. (2019). Environmental heterogeneity can tip the population genetics of range expansions. *eLife* 8, e44359. doi:10.7554/eLife.44359.
